# Characteristics of Pulmonary Inflammation in Patients with Different Forms of Active Tuberculosis

**DOI:** 10.3390/ijms252111795

**Published:** 2024-11-02

**Authors:** Galina S. Shepelkova, Vladimir V. Evstifeev, Yuriy S. Berezovskiy, Anush E. Ergeshova, Ruslan V. Tarasov, Mamed A. Bagirov, Vladimir V. Yeremeev

**Affiliations:** 1 Central Tuberculosis Research Institute, Moscow 107564, Russia; vladimir-evstifeev@yandex.ru (V.V.E.); report-q@yandex.ru (Y.S.B.); anush.ergeshova@mail.ru (A.E.E.); etavnai@yandex.ru (R.V.T.); m.bagirov@ctri.ru (M.A.B.); 2 Moscow Regional Clinical Tuberculosis Center, Mytishchi 141132, Russia

**Keywords:** active tuberculosis, immunopathogenesis, inflammatory reactions, miRNA, gene expression, cytokine production

## Abstract

Targeted treatment of tuberculosis-associated lung damage requires an understanding of the precise mechanisms of immunopathology. A major obstacle to the longitudinal study of tuberculosis (TB) immunopathogenesis in humans is the lack of serial lung biopsies during disease progression and treatment, which could be used to characterize local immune pathways involved in tissue damage. Understanding of the immunobiology of lung tissue damage in tuberculosis has largely been based on animal models. Our study looked for signs of inflammation in TB patients’ lung biopsies. Results were compared between a site of infection and relatively healthy tissue outside the site. The most significant differences in the expression of microRNAs (miRs) and cytokine/chemokines were observed between the non-decayed tuberculoma and the surrounding parenchyma. In addition, these parameters showed almost no differences between the cavitary wall and surrounding tissue. This is an indication that the inflammatory process is more prevalent in fibrotic cavitary tuberculosis (FCT). In FCT subjects, no difference was observed between the cavity wall and the parenchyma in the production of key inflammatory factors such as IL-6, IL-11, IL-17, and IFNγ. This is an indication that the limits of the inflammatory response are broader in FCT. The expression levels of miR-191, miR-193a, miR-222, miR-223, miR-18, miR-155, miR-376c, miR-26a, miR-150, and miR-124 were not significantly different between the cavernous wall and lung tissue in patients with FCT, further confirming the spread of inflammatory and destructive processes beyond the focus of infection.

## 1. Introduction

Pulmonary involvement in tuberculosis is remarkably heterogeneous. Formal pulmonary function tests measure levels of lung function, ranging from no impairment to severe dysfunction [1]. Patients may have cavitation, fibrosis, nodular infiltrates, or a mixture of these [2]. This enormous variability may be related to host–pathogen interactions and the multiple immunological events that may ensue. In addition, we suspect that the heterogeneity of lung injury is due in part to variations in genes that encode or regulate the host immune response. Therapies that specifically target immunological factors responsible for lung injury could be developed by understanding the immune pathways and genetic risk factors for TB-associated lung injury.

Still, most of what we know about how TB modulates the host epigenome is based on *in vitro*, small animal, or blood cell models, which do not accurately reflect the lung nature of the disease.

Published data analysis suggests that inflammation plays a dual role in the host’s response to mycobacteria. On the one hand, the process of granuloma formation limits the size of the focus of infection and dissemination. On the other hand, a violation of the inflammation control might lead to lung tissue damage, disease progression, and deterioration of the patient’s condition. Uncontrolled inflammatory reactions become pathogenic rather than protective. Primary mechanisms of TB progression may include a genetically determined inability of the host to control the replication of mycobacteria, overproduction of inflammatory cytokines and chemokines by cells, and much more.

A granuloma is a specialized structure in which T lymphocytes activate infected macrophages, resulting in inhibition of *Mycobacterium tuberculosis* growth and a decrease in the rate of dissemination of the infection. The main mediators of this process are the cytokines of the TNF family (TNF and LTα), IL-1α, and IL-1β, as well as the chemokines MCP-1, IP-10, and Mip-1α [3,4]. The production of all these molecules induces the migration of monocytes and lymphocytes into the infection zone and their aggregation with the formation of granulomas [5]. Usually, a dense granuloma is formed, in which dormant MBT persists for a long time. For a more reliable delimitation of the contents, a fibrous capsule is formed around the granuloma. The formation of the central necrotic zone and the outer layers and lymphocyte follicles complete the granuloma maturation processes. It is important to note that human tuberculous granuloma is characterized by a clear structure with central necrosis, surrounded by coaxial layers and follicles of cells of the immune system, separating necrosis from functioning lung tissue. Further intensification of inflammation leads to caseation and, as a consequence, the formation of cavities.

There are very few publications that correlate the varying activity of destructive and inflammatory processes in different forms of active pulmonary TB with the level of miRs expression. In our study, we evaluated the differences in miRs expression profiles in groups of surgical TB patients (tuberculoma without decay; tuberculoma with decay; FCT) and correlated them with the activity and prevalence of the TB process. Previously, we identified a pattern of six miRs (miR-155, miR-191, miR-223, miR-26a, miR-222, miR-320) [6], the expression level and direction of which in the serum of patients can be used to characterize changes in the level of destruction and activity of inflammatory processes in surgical patients with pulmonary TB.

The use of the surgical method makes it possible to increase the efficiency of treatment of patients with pulmonary TB up to 90–98% [7,8]. In Russia, operations for limited forms of pulmonary tuberculosis predominate in the pool of phthisiosurgical interventions. In particular, surgery for tuberculomas comprises 76.5% [7,8].

Although the mediators and signaling mechanisms that trigger and prolong the inflammatory response were thoroughly investigated, relatively little is known about the factors that control the chronic inflammatory process, as well as the factors responsible for the transition of chronic infection to the acute phase. Our work aimed to clarify these questions.

## 2. Results

### 2.1. Histological Picture of Post-Primary TB Lesions

**Figure 1:**
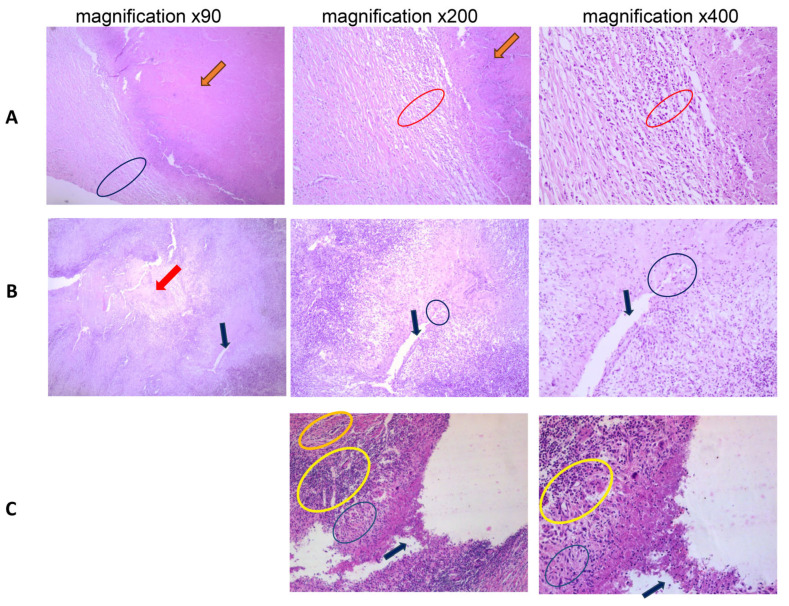
Histology of the focus of infection in the lung tissue of patients diagnosed as (**A**) tuberculoma without “decay”; (**B**) tuberculoma with “decay”; (**C**) cavitary (FCT). Hematoxylin and eosin stain. Magnification ×90; ×200; ×400. (**A**) Orange arrow—compacted caseose without inflammatory infiltration; blue oval—bi-layered fibrous capsule; red oval—layer in the fibrous capsule with a minimal amount of lymphomacrophage infiltrate without leukocytes. (**B**) Red arrow—area of caseous necrosis; black arrow—draining bronchus; blue circle—area of bronchial wall arrosion due to TB inflammation. (**C**) Black arrow—remnants of caseous masses in the cavernous lumen with the presence of inflammatory infiltrate; yellow oval—granulomatous layer of the cavernous lumen; blue oval—fibrous layer of the cavernous lumen.

Patients with active pulmonary tuberculosis were subject to pathomorphological examination at baseline. Typical images of tuberculomas and cavities of patients included in this study are shown in Figure 1.

In patients with tuberculoma without decay, foci of compacted caseous necrosis were surrounded by a mature double-layered connective tissue capsule with minor lymphocyte infiltration (Figure 1A).

Unlike tuberculoma without decay, tuberculoma with decay was characterized by a zone of “loose” caseosis with the formation of a central caseous-free zone (Figure 1B). This zone is the result of a bronchus entering the tuberculoma and draining the focus of infection (washing out the caseous masses). Large numbers of leukocytes and macrophages with lymphocyte admixture were observed in caseosis. The wall of the draining bronchus showed a granulomatous reaction with epithelioid cells, a high percentage of leukocytes, and a pronounced lymphoid shaft. The granulomatous reaction spread to the entire wall thickness of the draining bronchus, causing bronchial wall erosion in some locations. There were three layers in the decayed tuberculoma capsule surrounding the necrotic zone:

- A pyogenic layer dominated by macrophages and leukocytes (the main cause of the caseous liquefaction). This layer is formed at the expense of the vascularized granulation tissue.
- Mature connective tissue with an abundance of granulomatous reaction (multiphase plumose).
- Scar connective tissue with lymphoid infiltration and admixture of macrophages, adjacent to non-infected tissue.

In the majority of cases, the presence of a draining bronchus was characteristic of the cavity in patients with a diagnosis of fibrotic cavitary tuberculosis (FCT) (Figure 1C). The epithelium was eroded by granulomatous inflammation. Areas of squamous dysplasia were noted in the remaining bronchial epithelium.

The cavitary capsule consisted of three layers:

- Pyogenic layer, represented by irregularly expressed granulation tissue with leukocytic–lymphoid infiltrate mixed with macrophages;
- Granulomatous layer, a layer of fused granulomatous reaction within granulation tissue;
- Connective tissue layer adjacent to the parenchyma.

In the adjacent lung tissue, there were areas of caseating granulomatous inflammation and diffuse focal lymphoid infiltration. In the nearby inter-alveolar septa, there was a diffuse lymphoid infiltrate of varying degrees of severity. Macrophages with a small admixture of leukocytes were present in the alveolar lumen. Hyperplasia of the muscular layer, sclerosis, and areas of endothelial proliferation were observed in the vessels adjacent to and bordering the inflammatory zone. In some vessels, the lumen was obliterated.

### 2.2. Assessment of Inflammatory Status in Pulmonary Tissue from Patients with Active Tuberculosis

In patients with different forms of active tuberculosis, we compared inflammation directly at tuberculosis foci (tuberculoma/cavitary walls) with healthy tissue (parenchyma) adjacent to these foci. The volume fraction of tuberculoma or cavern tissue in the resected piece of lung is approximately 25%, with the remainder of the volume being in relatively intact tissue [9]. This allowed us to compare chemokine/cytokine gene expression in affected and relatively intact tissues.

**Figure 2:**
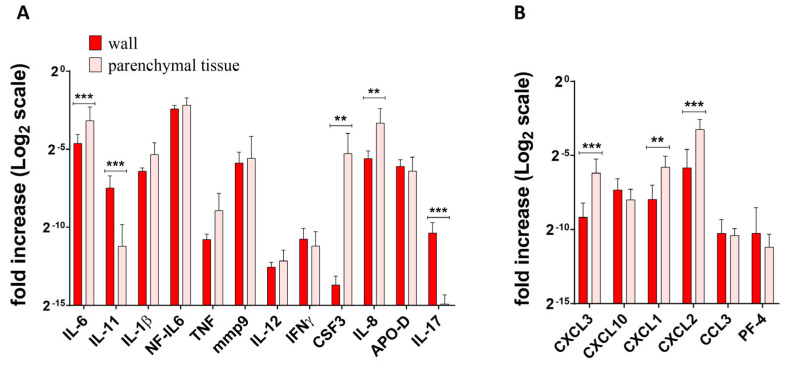
Gene expression profile of inflammatory factors (**A**) and chemokines (**B**) in lung tissue from patients diagnosed with FCT. RNA was isolated from biopsy surgical specimens (cavernous wall and healthy parenchymal tissue, as far away from the focus of infection as possible within the extent of the surgery) of patients diagnosed with FCT. From the RNA, cDNA was prepared and used as a matrix for QRT-PCR. GAPDH was used as a housekeeping gene. Means ± SD are shown (*n* = 50 individuals per group). **—*p* ˂ 0.01; ***—*p* ˂ 0.001.

**Figure 3:**
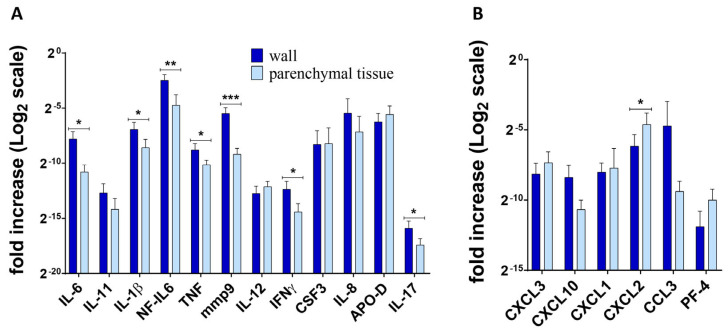
Gene expression profile of inflammatory factors (**A**) and chemokines (**B**) in lung tissue from patients diagnosed with tuberculoma with “decay”. RNA was isolated from biopsy surgical specimens (tuberculoma wall and healthy parenchymal tissue, as far away from the focus of infection as possible within the extent of the surgery) of patients diagnosed with FCT. From the RNA, cDNA was prepared and used as a matrix for QRT-PCR. GAPDH was used as a housekeeping gene. Means ± SD are shown (*n* = 50 individuals per group). *—*p* ˂ 0.05; **—*p* ˂ 0.01; ***—*p* ˂ 0.001.

**Figure 4:**
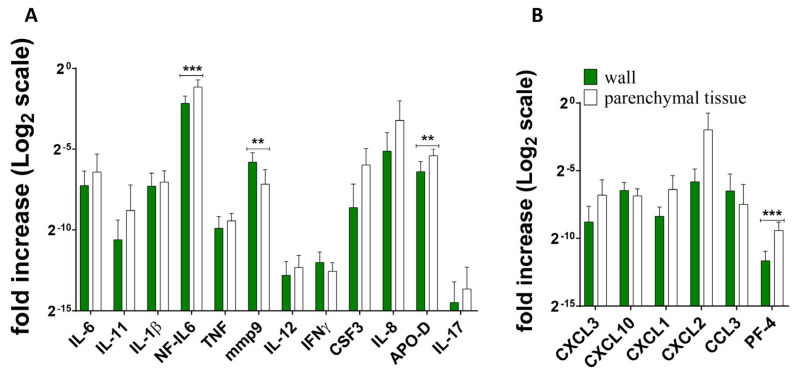
Gene expression profile of inflammatory factors (**A**) and chemokines (**B**) in lung tissue from patients diagnosed with tuberculoma without “decay”. RNA was isolated from biopsy surgical specimens (tuberculoma wall and healthy parenchymal tissue, as far away from the focus of infection as possible within the extent of the surgery) of patients diagnosed with FCT. From the RNA, cDNA was prepared and used as a matrix for QRT-PCR. GAPDH was used as a housekeeping gene. Means ± SD are shown (*n* = 50 individuals per group). **—*p* ˂ 0.01; ***—*p* ˂ 0.001.

Figure 2, Figure 3 and Figure 4 show the differences in cytokine/chemokine gene expression levels in the tuberculoma/cavitary wall with parenchymal lung tissues for each of the experimental groups. Comparison of cavernous wall with parenchymal tissues of FCT patients revealed the following differences: downregulated—IL-6, CSF3, CXCL3, CXCL1, CXCL2, IL-8; upregulated—IL-11, IL-17 (Figure 2); downregulated—CXCL2; upregulated—IL-6, IL-1β, IL-17, mmp9, IFNγ, TNF, and NF-IL6 in lesion wall and parenchymal tissue of tuberculoma with “decay” patients (Figure 3). Comparison of lesion wall with the parenchyma of tuberculoma without “decay” patients showed downregulation of PF-4, NF-IL6, and APO-D and upregulation of mmp9 (Figure 4).

**Figure 5:**
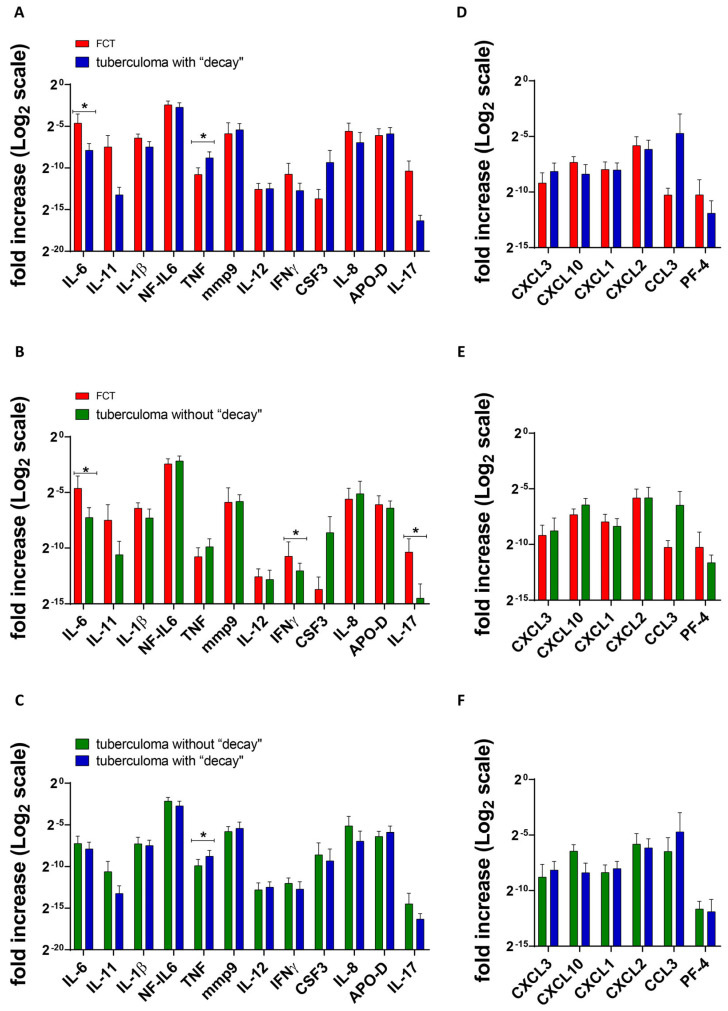
Gene expression profile of inflammatory factors (**A**–**C**) and chemokines (**D**,**E**) in tuberculoma/cavernous wall. FCT patients’ gene expression versus lung tuberculoma with “decay” (**A**,**D**); FCT patients’ gene expression versus lung tuberculoma without “decay” (**B**,**E**); lung tuberculoma with “decay” gene expression versus lung tuberculoma without “decay” (**D**,**F**). Means ± SD are shown (*n* = 50 persons per group). *—*p* < 0.05.

The corresponding tissues from patients in different groups were compared in a similar manner. Comparing the cavernous wall of FCT patients with the lesion wall of tuberculoma with “decay” patients, the following differences were observed: downregulated—TNF; upregulated—IL-6 (Figure 5A). The following differences were observed: downregulated—none; upregulated—IL-6, IL-17, and IFNγ when the cavernous wall of FCT patients was compared with the lesion wall of tuberculoma without “decay” patients (Figure 5B).

When lesion walls from tuberculoma with “decay” patients were compared with those of tuberculoma without “decay” patients, the following differences were marked: downregulated—CXCL10; upregulated—TNF (Figure 5C).

**Figure 6:**
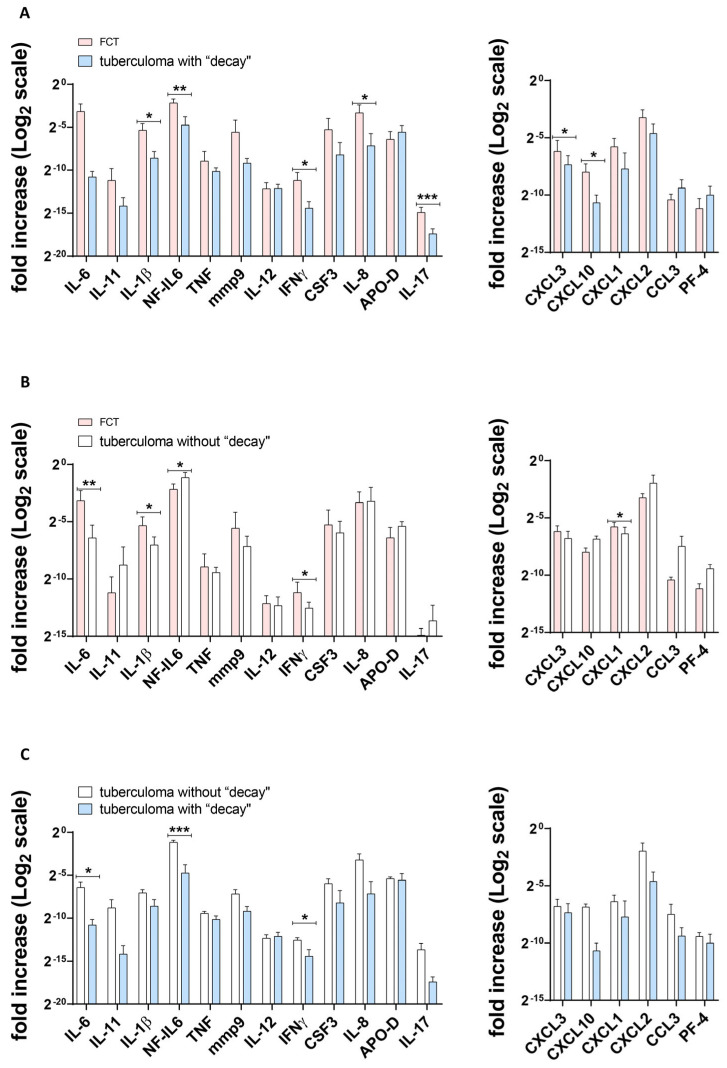
Gene expression profile of inflammatory factors and chemokines (**A**–**C**) in healthy lung parenchymal tissue, as far away from the focus of infection as possible within the extent of the surgery. FCT patients’ gene expression versus lung tuberculoma with “decay” (**A**); FCT patients’ gene expression versus lung tuberculoma without “decay” (**B**); lung tuberculoma with “decay” gene expression versus lung tuberculoma without “decay” (**C**). Means ± SD are shown (*n* = 50 persons per group). *—*p* < 0.05; **—*p* ˂ 0.01; ***—*p* ˂ 0.001.

The following differences were noted when comparing parenchymatous tissue from FCT patients with that from tuberculoma patients with “decay”: downregulated—CCL3; upregulated—IL-1β, IL-17, IFNγ, CXCL10, IL-8, NF-IL6 (Figure 6A).

Comparing parenchymatous tissues from FCT patients with those from tuberculoma patients without “decay”, the following differences were noted: downregulated—CXCL1, NF-IL6; upregulated—IL-6, IL-1β, IFNγ (Figure 6B).

Next, we compared the parenchymatous tissue of tuberculoma patients with “decay” with that of tuberculoma patients without “decay”: downregulated—IL-6, IFNγ, NF-IL6; upregulated—none (Figure 6C).

**Figure 7:**
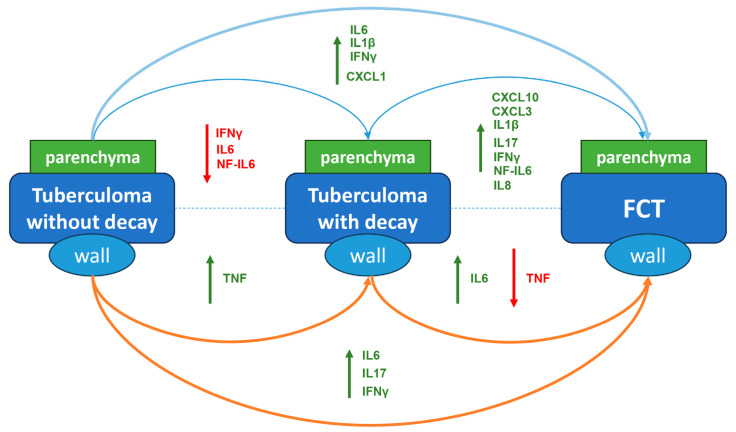
Cytokine and chemokine gene expression changes in tuberculoma/cavity wall (brown arrow) and surrounding parenchymal tissue (blue arrow) between groups of patients with different types of active TB. Red—downregulated genes; green—upregulated genes.

Overall, the comparative changes in cytokine and chemokine gene expression profiles are summarized in Figure 7.

**Figure 8:**
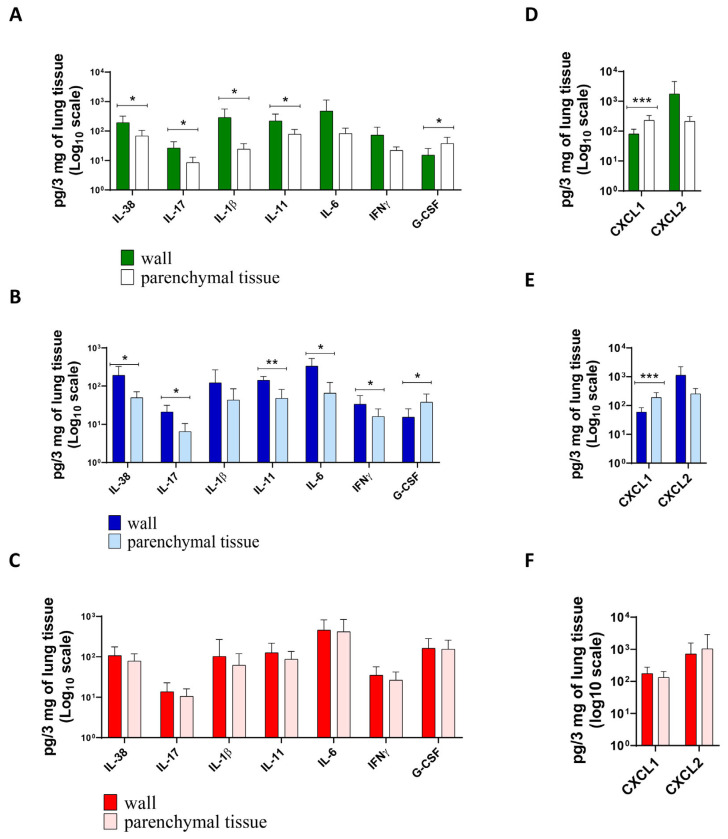
Pulmonary tissue production of inflammatory agents (**A**–**C**) and chemokines (**D**–**F**) in patients with tuberculoma without “decay” (**A**,**D**), tuberculoma with “decay” (**B**,**E**), and FCT (**C**,**F**). A total of 3 mg of lung tissue surgical biopsy material (from tuberculoma/cavitary wall and “healthy” parenchymal tissue) was homogenized in 3 mL PBS. The concentration of cytokines, chemokines, and inflammatory factors was determined by ELISA. Means ± SD are shown (*n* = 50 patients in each group). *—*p* < 0.05; **—*p* < 0.01; ***—*p* < 0.001.

The data on the production of inflammatory factors in the lung tissue of patients with different forms of lung TB agreed with the expression data and confirmed the differences in the level of inflammatory factors in the different groups. Patients diagnosed with tuberculoma without “decay” had the lowest level of inflammation (Figure 8A,D). Patients diagnosed with tuberculoma with “decay” showed increased inflammation. Compared to “healthy” lung parenchyma (Figure 8B), the concentration of proinflammatory cytokines IL-6, IL-11, IL-17, and IFNγ, which are responsible for granuloma organization and infection focus delineation, was increased in the tuberculoma wall. The levels of the proinflammatory cell migration factors G-CSF and CXCL1 were significantly higher in “healthy” lung tissue (Figure 8B,E). No difference in the production of key inflammatory factors was observed between the cavitary wall and parenchyma in FCT subjects (Figure 8C,F). This observation is suggestive of a broadening of the boundaries of the inflammatory response. In the lung tissue of patients with FCT, the inflammatory process is not confined to the cavernous wall, but spreads to the “healthy” parenchymal lung tissue as the infection spreads.

### 2.3. microRNA Gene Expression in Inflamed and Peripheral Tissues of Post-Primary TB Lungs

**Figure 9:**
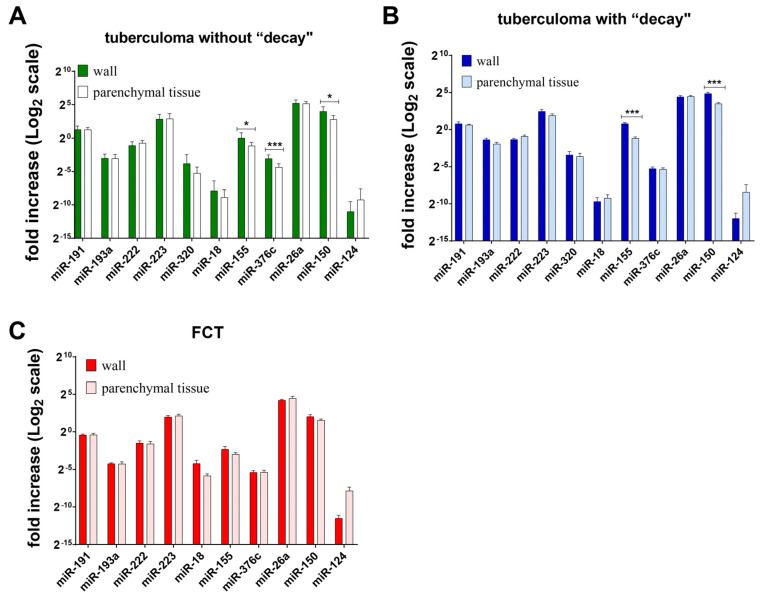
Expression of genes encoding mature miRNAs in the lung tissue of active lung TB patients. (**A**) miRNA expression of lung tuberculoma without “decay” patients; (**B**) miRNA expression of lung tuberculoma with “decay” patients; (**C**) miRNA expression of FCT patients. RNA was isolated from biopsy surgical specimens (tuberculoma wall and healthy parenchymal tissue, as far away from the focus of infection as possible within the extent of the surgery) of active TB patients. From the RNA, cDNA was prepared and used as a matrix for QRT-PCR. Means ± SD are shown (*n* = 50 patients per group). *—*p* < 0.05; ***—*p* < 0.001.

Our studies showed that the expression level of inflammation-related miRs is higher in the tuberculoma wall than in “healthy” lung tissue (Figure 9A,B). In the tuberculoma wall, miR-155, miR-376c, and miR-150 expression levels were increased in patients diagnosed with tuberculoma without “decay” (Figure 9A); miR-155 and miR-150 expression levels were increased in patients diagnosed with tuberculoma with decay (Figure 9B). In patients with FCT, the expression levels of the miRs identified were not significantly different in the cavernous wall and lung tissues (Figure 9C), which may provide additional confirmation that inflammatory and destructive processes spread beyond the focus of infection.

**Figure 10:**
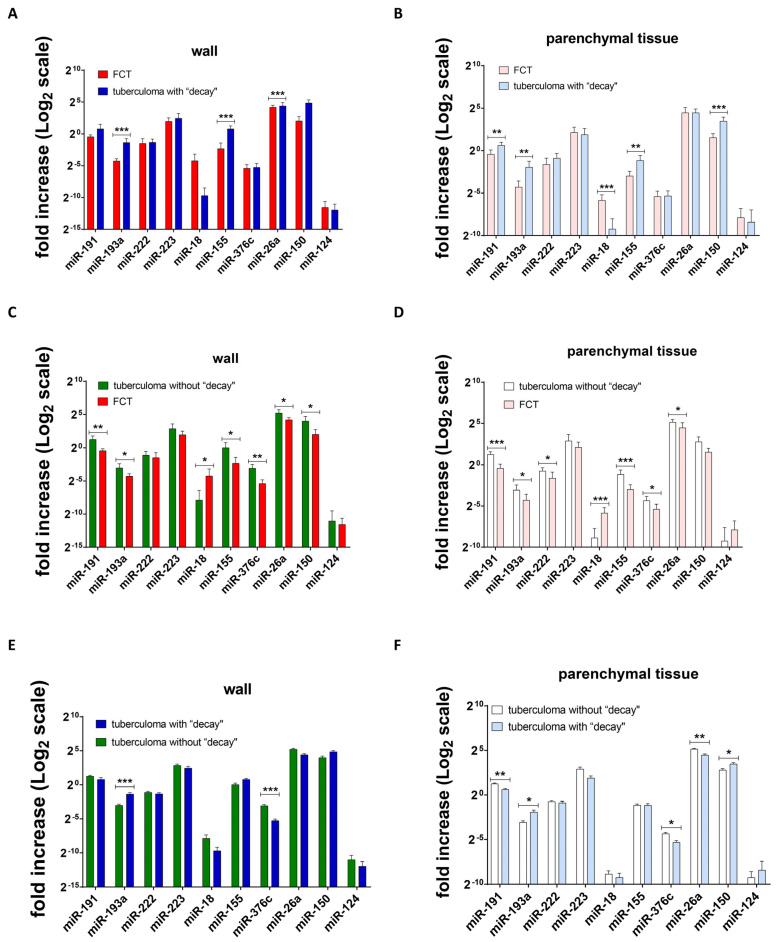
Differences in the expression of miRNAs in the lung tissue of patients with different types of active tuberculosis. (**A**) FCT patients’ miRNA expression versus lung tuberculoma with “decay” patients’ miRNA expression (tuberculoma/cavernous wall); (**B**) FCT patients’ miRNA expression versus lung tuberculoma with “decay” patients’ miRNA expression (healthy parenchymal tissue, as far away from the focus of infection as possible within the extent of the surgery); (**C**) FCT patients’ miRNA expression versus lung tuberculoma without “decay” patients’ miRNA expression (tuberculoma/cavernous wall); (**D**) FCT patients’ miRNA expression versus lung tuberculoma without “decay” patients’ miRNA expression (healthy parenchymal tissue, as far away from the focus of infection as possible within the extent of the surgery); (**E**) lung tuberculoma without “decay” patients’ miRNA expression versus lung tuberculoma with “decay” patients’ miRNA expression (tuberculoma/cavernous wall); (**F**) lung tuberculoma without “decay” patients’ miRNA expression versus lung tuberculoma with “decay” patients’ miRNA expression (healthy parenchymal tissue, as far away from the focus of infection as possible within the extent of the surgery). Means ± SD are shown (*n* = 50 patients per group). *—*p* < 0.05; **—*p* < 0.01; ***—*p* < 0.001.

MiRNA expression levels were also compared in patients with different forms of active TB separately in the tuberculoma/cavernous wall and in “healthy” lung tissue. When the caverna wall from FCT patients was compared with the lesion wall from tuberculoma with “decay” patients, the following differences were marked: downregulated—miR-193a, miR-155, miR-26a; upregulated—none (Figure 10A).

When the caverna wall from FCT patients was compared with the lesion wall from tuberculoma without “decay” patients, the following differences were marked: downregulated—miR-191, miR-193a, miR-155, miR-376c, miR-26a, miR-150; upregulated—miR-18 (Figure 10C).

Next, we compared the wall tissue of tuberculoma patients with “decay” with that of tuberculoma patients without “decay”: downregulated—miR-376c; upregulated—miR-193a (Figure 10E).

In the case of comparison of miRNA expression levels in “healthy” parenchymatous lung tissue, differences have also been noted.

The following differences were noted when comparing parenchymal tissue from FCT patients with that from tuberculoma patients with “decay”: downregulated—miR-191, miR-193a, miR-155, miR-150; upregulated—miR-18 (Figure 10B).

Comparing parenchymal tissues from FCT patients with those from tuberculoma patients without “decay”, the following differences were noted: downregulated—miR-191, miR-193a, miR-222, miR-155, miR-376c, miR-26a; upregulated—miR-18 (Figure 10D).

When parenchymatous tissue from tuberculoma with “decay” patients was compared with that of tuberculoma with “decay” patients, the following differences were marked: downregulated—miR-191, miR-376c, miR-26a; upregulated—miR-193a, miR-150 (Figure 10F).

**Figure 11:**
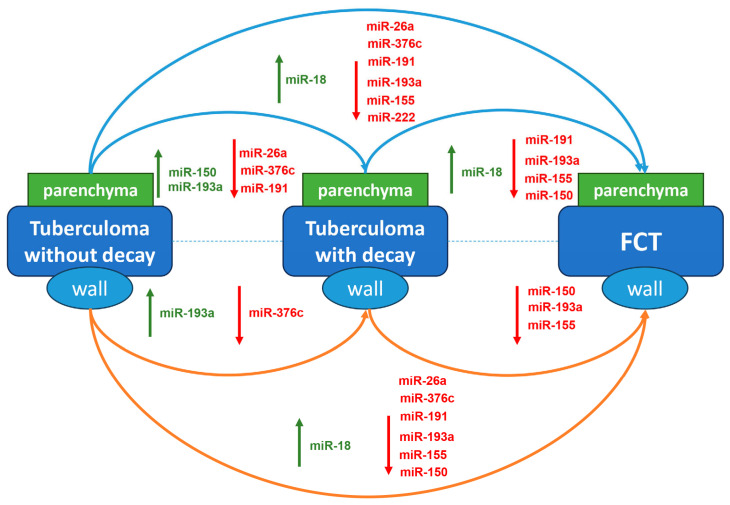
miR gene expression changes in tuberculoma/cavity wall (brown arrow) and surrounding parenchymal tissue (blue arrow) between groups of patients with different types of active TB. Red—downregulated genes; green—upregulated genes.

Overall, the comparative changes in miR gene expression profiles are summarized in Figure 11.

## 3. Discussion

Based on the data obtained from histology studies, it can be assumed that there is minimal development of inflammatory responses in the focus of infection of patients with tuberculoma without decay. As the process progresses and decay occurs, there is an increased development of inflammation. Leukocyte and macrophage migration into the infection focus increases. A rich granulomatous reaction develops in the tuberculoma wall and infiltrates healthy lung tissue (Figure 1B). Inflammation tends to peak in patients diagnosed as having FCT. In these patients, inflammation extends beyond the focus of infection to the surrounding parenchymal tissue (Figure 1C). Cavitary lesions are clearly associated with TB progression and spread, whereas granulomas have a dual protective and pathological role. Cavitary lesions present a thin layer of caseous necrosis containing predominantly neutrophils [10] and macrophages, including epithelioid and giant cells. Such lesions are rich in bacteria [11,12]. These appear as pellicles on the cavity wall [13], from which *M. tuberculosis* is expectorated. Radiological studies of patients with active tuberculosis show that the presence of cavities correlates with an increase in the number of bacilli in the sputum [14]. Cavities reduce the antimicrobial effectiveness of TB drugs and are associated with the development of antibiotic resistance and treatment failure [15,16,17]. Cavities result from excessive inflammation. According to the current paradigm, cavities result from the cavitation of caseous granulomas [17]. A local imbalance of inflammatory processes leads to extensive necrosis of the granuloma and leakage of its liquefied contents into the bronchi.

The data on the production of inflammatory factors in the lung tissue of patients with different forms of lung TB agreed with the expression data and confirmed the differences in the level of inflammatory factors in the different groups. Patients diagnosed with tuberculoma without “decay” had the lowest level of inflammation (Figure 8A,D). Patients diagnosed with tuberculoma with “decay” showed increased inflammation. Compared to “healthy” lung parenchyma (Figure 8B), the concentration of proinflammatory cytokines IL-6, IL-11, IL-17, and IFNγ, which are responsible for granuloma organization and infection focus delineation, was increased in the tuberculoma wall. The levels of the proinflammatory cell migration factors G-CSF and CXCL1 were significantly higher in “healthy” lung tissue (Figure 8B,E). No difference in the production of key inflammatory factors was observed between the cavity wall and parenchyma in FCT subjects (Figure 8C,F). This suggests that the limits of the inflammatory response are extended. The inflammatory process is not limited to the cavitary wall but spreads to the “healthy” parenchymal lung tissue as the infection progresses in the lung tissue of patients with FCT.

Increased APO-D expression (Figure 4A) may indicate the presence of macrophages at the site of infection, which may contribute to the structuring of granulomas and the demarcation of inflammatory processes from “healthy” lung tissue. The presence of a platelet reaction is indicated by increased expression of PF-4, which is produced by activated platelets. By forming aggregates with immune cells and releasing chemokines and growth factors, platelets can mediate certain immunological mechanisms [18]. In TB, platelets are involved in granuloma formation by producing chemokines to recruit innate immune cells including monocytes and macrophages. Platelets also have the ability to induce a monocyte profile with strong collagenase activity and to induce monocytes to differentiate into multinucleated giant cells [19,20]. Furthermore, PF-4 is involved with the migration of CCR1^+^ monocytes [21], which could be an important mechanism during Mtb infection.

In a previous study, we showed that miR-155, miR-191, and miR-223 were differentially expressed in sera of “decaying” and “non-decaying” tuberculomas. A further group (miR-26a, miR-191, miR-222, and miR-320) discriminated tuberculomas with “decay” from FCT. The serum expression of miR-26a, miR-155, miR-191, miR-222, and miR-223 in tuberculomas not diagnosed as “decay” differs from that in tuberculomas with FCT [6]. Here, the expression of TB-related miRs in the lung tissue of TB patients was compared using the same set of markers comprising ten miRNAs (miR-191, miR-193a, miR-222, miR-223, miR-18, miR-155, miR-376c, miR-26a, miR-150, and miR-124).

Analysis of the expression of mature miRs also revealed an increase in destructive and inflammatory processes in lung tissue in the direction of tuberculoma without decay → tuberculoma with decay → FCT. The expression of TB inflammation-related miRs was shown to be higher in the tuberculoma wall than in “healthy” lung tissue (Figure 9A,B). As an additional confirmation of the spread of inflammatory and destructive processes beyond the focus of infection, the expression level of miRs did not differ significantly between the cavernous wall and lung tissue in patients with FCT (Figure 9C).

The dynamics of miRNA expression in the lungs of *M. tuberculosis* patients remain unknown, whereas miRNA profiling has been performed mainly in the blood or serum of TB patients or in specific cells such as macrophages [22]. In order to gain insight into this process, we determined the miRNA expression signatures in inflamed versus intact pieces of lungs isolated from Mtb-infected individuals and identified an altered expression pattern for several miRs. Our study suggests that different levels of miRs may influence different molecular pathways and contribute to infection progression.

## 4. Materials and Methods

### 4.1. Patients and Control Subjects

This study included 150 patients with active pulmonary TB from the Surgical Department of the Central Tuberculosis Research Institute (Moscow, Russia). The patients aged 18–65 years (69 women and 81 men) with clinical and radiological findings consistent with active pulmonary TB, positive acid-fast mycobacterial growth, and/or a positive gene-Xpert^®^ test were enrolled from 1 January 2017 to 31 December 2021. Exclusion criteria included age less than 18 years, pregnancy, history of diabetes, multiple allergies, asthma, systemic autoimmune disorders, active infection, chronic heart failure, acute myocardial infarction, and continuous use of corticosteroids. All patients received pre- and postoperative chemotherapy for TB. According to chest computed tomography (CT) data, patients were divided into the following groups:

- Patients who were diagnosed with pulmonary tuberculoma without “decay” (50 persons);
- Patients who were diagnosed with lung tuberculoma with “decay” (50 persons);
- Patients who were diagnosed with FCT (50 persons).

Biopsies of lung tissue (D = 1 cm) were obtained from each patient (from the focus of infection, i.e., the wall of the tuberculoma or cavern, and from relatively healthy lung tissue as far away from the focus of infection as possible within the extent of surgery). The Declaration of Helsinki was followed in all procedures and in the overall design of the study. This study had the approval of the Institutional Ethics Committee (IEC) of the Central Tuberculosis Research Institute (Moscow, Russia). Before enrollment, all participants were given a full explanation of the study’s objectives and processes.

### 4.2. Histology

Lung tissue sections were stained with hematoxylin and eosin (H&E) using a standard technique that is routinely used in the laboratory [23]. Histologic mount media was used to store the stained sections (Thermo Fisher Scientific (Thermo Shandon), Waltham, MA, USA). A Leica DMRB light microscope was used to take digital photographs of stained lung tissue sections (Leica Microsystems GmbH, Wetzlar, Germany).

### 4.3. Cytokine Expression Analysis

A total of 10 mg of lung tissue pre-fixed in RNase inhibitor solution, RNALater (Thermo Fisher Scientific, Waltham, MA, USA), was used for tissue RNA isolation. RNA isolation was performed with TRIzol^®^ Reagent (Thermo Fisher Scientific (Ambion), Waltham, MA, USA) in accordance with a previously described protocol [23]. SuperScript™ III Reverse Transcriptase (Thermo Fisher Scientific, Waltham, MA, USA) was used for reverse transcription. TaqMan™ Gene Expression Assay (FAM) (Thermo Fisher Scientific, Applied Biosystems, Waltham, MA, USA) was used for the determination of inflammatory factor gene expression by qRT-PCR. Data are presented as 2^ΔCt. GAPDH was used as a housekeeping gene.

### 4.4. Cytokine Production Analysis

Cytokine and chemokine concentrations in human lung tissue homogenates were measured by ELISA. DuoSet ELISA kits (R&D Systems, Minneapolis, MN, USA) for human IFNγ, IL-38, IL-17, IL-1β, IL-11, IL-6, G-CSF, CXCL1, and CXCL2 were used according to the manufacturer’s recommendations.

### 4.5. miR Expression Analysis

The TaqMan^®^ Advanced miRNA cDNA Synthesis Kit (Thermo Fisher Scientific (Applied Bio-systems), Waltham, MA, USA) was used. qRT-PCR for miRNA expression analysis (miR-191, miR-193a, miR-222, miR-223, miR-18, miR-155, miR-376c, miR-26a, miR-150, and miR-124) was performed according to a standard protocol [6]. miR-103 was selected as the reference for the analysis [24].

### 4.6. Statistical Analysis

Statistical analysis was performed using Prism 8.0.1 software. A multiple comparisons *t*-test (Sidak–Bonferroni method) was used to compare changes between groups; *p*-values less than 0.05 were considered significant in all analyses. The data are presented as the mean value ± SD (standard deviation of the mean).

## 5. Conclusions

Our results demonstrate spatial differentiation of inflammatory factor expression in lung tissue from patients with three different forms of active TB. The maximum differentiation of cytokine/chemokine expression as well as mature microRNAs is observed between the tuberculoma without decay wall and surrounding parenchyma. At the same time, there are virtually no differences in these parameters between the wall of the cavern and the surrounding tissue in patients with FCT. This suggests that inflammation is more widespread in FCT. Presumably, the use of anti-inflammatory therapy in combination with anti-tuberculosis therapy can be recommended at the stage of preparing the patient for surgical treatment.

## Data Availability

The data are unavailable due to privacy or ethical restrictions.
